# Disordered Eating Behaviors, Perceived Stress and Insomnia During Academic Exams: A Study Among University Students

**DOI:** 10.3390/medicina61071226

**Published:** 2025-07-06

**Authors:** Elena-Gabriela Strete, Mădălina-Gabriela Cincu, Andreea Sălcudean

**Affiliations:** 1 Department of Psychiatry, George Emil Palade University of Medicine, Pharmacy, Science and Technology of Târgu Mureş, 540142 Târgu Mureș, Romania; elena.buicu@umfst.ro; 2 Faculty of Medicine, George Emil Palade University of Medicine, Pharmacy, Science and Technology of Târgu Mures, 540142 Târgu Mureș, Romania; 3 Department of Ethics and Social Sciences, George Emil Palade University of Medicine, Pharmacy, Science and Technology of Târgu Mureș, 540142 Târgu Mures, Romania; andreea.salcudean@umfst.ro

**Keywords:** academic stress, insomnia, eating behaviors, students, university, exams

## Abstract

*Background and Objectives:* During exam sessions, many students experience high levels of stress caused by the large volume of material to study, tight deadlines, and pressure to achieve top grades. This study aimed to examine the relationship between academic stress, sleep disturbances, and eating behaviors by using validated questionnaires administered to a student sample. As stress levels increased, the data revealed a higher frequency of insomnia symptoms and disordered eating, including behaviors such as compulsive eating and irregular meal patterns. *Materials and Methods:* This cross-sectional, correlational study was conducted on a convenience sample of 317 university students from various academic centers across Romania. Participants were recruited via online distribution of a self-administered questionnaire during a four-month period, including exam sessions. The survey included the Perceived Stress Scale (PSS), the Insomnia Severity Index (ISI), and the Eating Disorder Examination Questionnaire (EDE-Q), alongside additional items assessing perceived links between stress, sleep, and eating behaviors, and the use of medication. Data were analyzed using SPSS with Spearman correlations, *t*-tests, and linear regression. *Results:* Statistical analyses revealed significant and positive associations between perceived stress levels and insomnia, as indicated by Spearman’s correlation (*p* < 0.01). A similar significant correlation was identified between perceived stress and disordered eating behaviors among students. Specifically, the feeling of being overwhelmed by academic workload showed a strong positive correlation with a lack of control over eating behaviors (r = 0.568). Furthermore, linear regression analysis confirmed a significant predictive relationship between feeling overwhelmed and the tendency to eat excessively beyond initial intentions, with a standardized regression coefficient B = 0.581 (*p* < 0.001). A separate regression analysis focusing on exam-related stress and episodes of compulsive eating behavior demonstrated a comparable result (B = 0.573, *p* < 0.001), indicating a robust positive association. Additionally, independent samples *t*-tests demonstrated that students experiencing high levels of stress during the exam period reported significantly greater difficulties with sleep initiation and higher levels of disordered eating compared to their peers with lower stress levels. The difference in insomnia scores was highly significant (*t* = 11.516, *p* < 0.001), as was the difference in eating behavior scores (*t* = 10.931, *p* < 0.001). *Conclusions:* These findings underscore the need for emotional support services and effective stress management strategies, enabling students to navigate academic demands without compromising their mental or physical well-being.

## 1. Introduction

While studying at a university, exam periods are often perceived as true tests of intellectual and emotional endurance. For most of the students, this phase of the academic process is not only about passing exams but also involves multiple stress factors that can disturb their psychological and biological balance.

Although academic stress is a well-recognized phenomenon in university environments, the magnitude of its consequences on daily behavior, sleep habits, and eating habits often remains insufficiently explored among the students in Romania. Furthermore, the specialized literature suggests that perceived stress levels among students can be influenced by factors such as regular physical activity and field of study. Students who engage in frequent physical exercise tend to manage academic stress more effectively due to the release of endorphins and improved sleep regulation [1,2,3,4,5,6,7,8,9,10,11]. Moreover, studies report significant differences between academic disciplines: medical students often experience higher levels of stress compared to those in the humanities, due to the intense academic workload, competitiveness, and demanding schedules [12]. These factors contribute to increased vulnerability to sleep disturbances and disordered eating behaviors during exam periods.

Recent studies from international literature confirm the close link between perceived stress and the occurrence of sleep disturbances, especially insomnia. This common phenomenon often appears in stressful contexts, even among young people [13,14]. It serves as a key mediating factor linking chronic stress with affective disorders such as depression and anxiety, underscoring the necessity of effectively managing these disturbances [15]. On a national level, studies have highlighted the biochemical implications of stress on mental health, accentuating serotonin changes and the neurophysiological effects of ongoing stress [16,17,18]. Specialized literature confirms that chronic stress can significantly affect both the circadian rhythm and an individual’s relationship with food. Recent studies have shown that prolonged stress often leads to issues like insomnia, alongside disordered eating behaviors such as binge eating or meal avoidance [19,20]. These behaviors are not just temporary responses—without proper intervention, they can become risk factors for long-term physical and psychological imbalances [21,22].

In the context of the exam period, these imbalances tend to increase. Recent studies published in medical journals have increasingly focused on the psychological impact of stress on vulnerable populations’ functioning, leading to a decline in quality of life, reduced adherence to medical treatments, and difficulties in maintaining healthy lifestyle habits [23,24,25]. Moreover, chronic stress can worsen sleep disturbances and increase vulnerability to psychiatric disorders, further compromising overall health [26]. This study aligns with these findings, offering a practical perspective on the way academic stress affects students’ sleep and eating habits.

The primary objective of this research is to investigate the relationship between the perceived stress levels during exams and the occurrence of sleep and eating disorders. Based on existing studies and clinical observations of students, we hypothesize that higher levels of academic stress are associated with an increase in symptoms of insomnia and disordered eating. We also considered that the interaction between stress, insomnia, and disordered eating creates a specific psychological vulnerability during exam periods [27,28].

The study is built around the following hypotheses:

**Hypothesis** **1.** *There is a significant positive association between perceived stress and the prevalence of insomnia.*

**Hypothesis** **2.** *Higher stress levels are associated with an increased frequency of eating disorders.*

**Hypothesis** **3.** *Insomnia acts as a mediator between stress and disordered eating.*

**Hypothesis** **4.** *Students experiencing higher stress levels are more likely to report both insomnia and eating disorders compared to those with lower stress levels.*

With this approach, the study aims to highlight the psychological challenges students face during exams and to help develop effective prevention and intervention strategies. In a society that increasingly values mental health as part of educational and professional success, research like this is essential to better understand the needs of young people in training.

## 2. Materials and Methods

**Figure 1:**
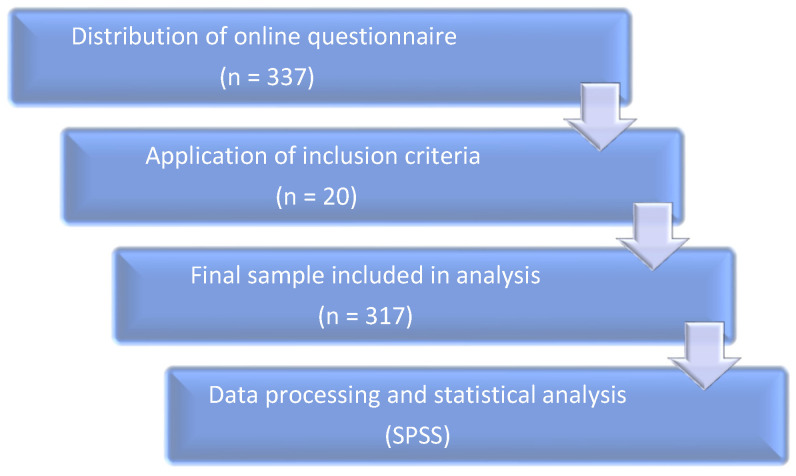
Flowchart illustrating the participant selection and data analysis process.

The research was conducted on a convenience sample of 317 students from various university centers across Romania. This process is summarized in Figure 1.

Inclusion criteria required participants to be enrolled as university students in Romania and to have completed the full questionnaire. Responses with missing or incomplete data were excluded from the final analysis. Out of the total 337 questionnaires initially received, 20 were excluded due to incomplete or inconsistent responses. Consequently, the final sample included 317 participants whose data met the inclusion criteria and were valid for analysis.

The study used a quantitative, cross-sectional design to investigate correlational relationships between academic stress, insomnia, and disordered eating behaviors over a four-month period (from December 2024 to March 2025), which included one exam session. Participants completed an online questionnaire composed of three adapted instruments: the Perceived Stress Scale (PSS) to measure levels of perceived stress, the Insomnia Severity Index (ISI) to assess sleep disturbances, and the Eating Disorder Examination Questionnaire (EDE-Q) to identify dysfunctional eating behaviors. The participants were selected using a non-probability, convenience sampling method, based on voluntary participation. The online questionnaire was distributed during the exam period via academic platforms, student groups, and social media channels, targeting students enrolled in various university centers across Romania.

Additional questions explored the perceived relationship between stress, insomnia, and eating difficulties, as well as the use of medication to induce sleep or relieve stress during the exam period. The collected data were statistically analyzed using IBM SPSS Statistics, version 20, applying the Spearman correlation coefficient, *t*-tests, and linear regression. Participation was anonymous and voluntary, in accordance with the ethical principles of psychological research. The study was reviewed and approved by the Research Ethics Committee of the “George Emil Palade” University of Medicine, Pharmacy, Science, and Technology of Târgu Mureș, in accordance with Decision No. 3401 issued on 12 November 2024, confirming compliance with the ethical standards of scientific research.

## 3. Results

### 3.1. Demographic Characteristics of the Sample of Students

The sample consists of 317 students from various university centers in Romania. The ages of the respondents range from under 20 to over 25 years, and statistics show that the majority (41%) are between 20 and 22 years old, while those under 20 and those aged 23 to 25 each represent 25.9% of the sample. The gender distribution was 60.9% women, 39.1% men. Regarding the year of study, 55.83% were in their final years, and 44.17% in lower years. Most of them reported living in dormitories or rented accommodations (84.8%), while the rest live with family, in their own homes, or have other living arrangements.

### 3.2. The Level of Perceived Stress During the Exam Period

This section analyzes the level of stress experienced by students during the exam sessions, marked by the pressure of tests and deadlines. To evaluate this, the Perceived Stress Scale (PSS) questionnaire was used, adapted to the Romanian university context, to understand how students feel and manage academic stress.

As shown in Table 1, during the exam period, the highest perceived stress level was associated with exams themselves, showing a mean score of 3.997 (SD = 0.634). The next most significant stressor was the feeling of having too many responsibilities, with a mean of 3.984 (SD = 0.658). Students also reported feeling overwhelmed by their workload (M = 3.934) and struggling to manage important aspects of life (M = 3.902) as notable sources of stress. Fear of not meeting academic expectations had the lowest mean score (3.880), yet it remained a relevant concern.

### 3.3. Insomnia Symptoms and Their Relation to Academic Stress

This section aims to identify sleep-related difficulties of the students during the exams, such as problems falling asleep, night awakenings, or early morning awakenings. The questionnaire, adapted from the Insomnia Severity Index (ISI), allowed for the assessment of specific types of insomnia—onset, maintenance, and terminal—in correlation with the increased academic stress characteristic of the exam period.

As shown in Table 2, the results indicate a high level of sleep difficulties among students during an exam period, with the average score above 3.7 on a scale of 1 to 5, for all variables analyzed. The most common problems are severe fatigue and the perception of unrefreshing sleep, aspects closely linked to the stress generated by exams. The most noticeable difficulties students mentioned were persistent tiredness and a feeling of not sleeping enough, followed by worries related to exams. These findings underscore a clear impact of academic stress on sleep quality.

### 3.4. Dysfunctional Eating Behaviors Under Stress

In the context of academic stress experienced during exam periods, students’ eating behaviors can undergo significant changes. Some of the subjects report episodes of binge eating, meal skipping, or consumption of junk food (with too much sugar or fat). These behaviors may indicate serious emotional problems resulting from continuous pressure and a lack of effective stress management strategies.

As shown in Table 3, during the exam period, students reported several signs of disordered eating associated with academic stress. The highest mean score was observed for eating more than initially intended (M = 3.70), followed closely by binge eating episodes (M = 3.71) and a lack of control over eating (M = 3.69), suggesting a tendency toward impulsive food intake under pressure. Other notable behaviors included food avoidance as a method of weight control (M = 3.69), feelings of guilt or shame after eating (M = 3.65), and excessive exercise to compensate for eating (M = 3.62). Preoccupation with body weight and shape also scored high (M = 3.69), as did restrictive eating practices (M = 3.64). Overall, the findings indicate a clear connection between academic stress and unhealthy eating behaviors, with students resorting to both overconsumption and restriction as coping strategies.

### 3.5. The Relationship Between Stress, Insomnia, and Eating Disorders

Academic stress, frequently experienced during exams, has a significant impact on the psychophysiological balance of students, directly influencing both sleep quality and eating behaviors. Emotional issues related to stress can lead to difficulty falling asleep, frequent nighttime awakenings, and the feeling of restless sleep. These factors may contribute to dysfunctional eating behaviors, such as compulsive eating or meal avoidance. Exploring the relationship between stress, insomnia, and eating problems offers a more comprehensive understanding of how students react to academic pressure.

As shown in Table 4, the descriptive analysis highlights a significant association between academic stress, sleep disturbances, and students’ eating behaviors. Mean scores above 3.7 suggest that many students tend to use food as an emotional coping mechanism, leading to unhealthy eating patterns and episodes of compulsive consumption. Insomnia further disrupts appetite regulation and meal planning, contributing to weight fluctuations. These findings reinforce the interplay between stress, sleep, and nutrition, pointing to potential risks for both physical and psychological well-being during exam periods.

### 3.6. Using Sleep and Stress Medication During Exams

The use of sleep- and stress-related medication during exam periods represents a coping mechanism for students facing academic pressure. In response to persistent stress, insomnia, and fatigue, some students resort to pharmacological solutions to maintain daily functioning. This section explores the frequency and motivations behind the use of such products, while also highlighting the potential long-term risks associated with self-medication. 

As shown in Table 5, although students reported high levels of stress and sleep disturbances during exam periods, their use of medication remained low. The average score for using sleep aids or medication to reduce stress was M = 1.85, while natural supplements had a slightly higher mean of 1.89. Prescription medication showed the lowest usage (M = 1.35), and the perceived need for sleep medication remained low as well (M = 1.88). These results suggest that, despite experiencing significant difficulties, most students avoid pharmacological coping strategies.

## 4. Discussions on Confirming the Hypotheses Through Variable Correlation

To test the hypotheses formulated, we used Spearman correlations, linear regressions, and mean comparison tests in the SPSS program. This approach allows us to understand how these variables influence one another, offering a clear perspective on the impact of stress on the psychological and behavioral balance of students.

**Hypothesis** **1:** *A significant positive association is assumed between perceived stress and the prevalence of insomnia.*

To test Hypothesis 1, we used the Spearman correlation coefficient. We analyzed the correlations between stress levels and insomnia scores obtained from the students’ responses to the questionnaire. This method allowed us to determine whether a significant direct correlation exists between the two variables.

**Figure 2:**
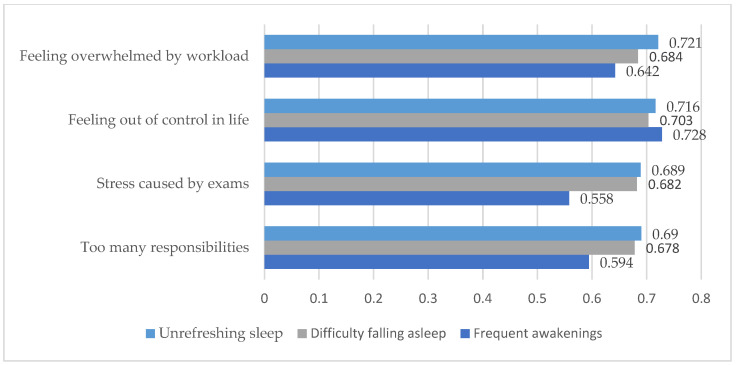
Main correlations between academic stress and insomnia (Spearman’s rho).

As shown in Table 6, the main correlations between academic stress and insomnia were also illustrated in graphical form in Figure 2 to enhance clarity and facilitate interpretation of key findings.

As shown in Table 6 and Figure 2, the Spearman correlation results revealed statistically significant and positive associations (*p* < 0.001) between perceived stress levels and various forms of insomnia. The strongest correlations were found between the feeling of losing control in life (I_6) and frequent nighttime awakenings (r = 0.728), as well as between feeling overwhelmed by workload (I_5) and experiencing unrefreshing sleep (r = 0.721). These high correlation coefficients suggest that as perceived stress increases, sleep difficulties also intensify, thereby supporting the hypothesis of a clear link between academic stress and insomnia.

**Hypothesis** **2:** *A high level of stress is associated with an increase in the frequency of eating disorders.*

To test the hypothesis regarding the relationship between stress and disordered eating, we used Spearman’s correlation coefficient and linear regression analysis. These methods allowed us to examine both the direction and strength of the association, providing insight into how stress influences students’ eating behaviors.

The Spearman correlation analysis confirmed significant relationships between the stress levels perceived by students and their unbalanced eating behaviors. For example, the feeling of being overwhelmed by the workload strongly correlates with a lack of control over eating (r = 0.568) and with episodes of compulsive eating (r = 0.525). Stress caused by exams shows a significant association with avoiding food for weight control (r = 0.487) and eating more than initially intended (r = 0.443). Additionally, the fear of not meeting academic demands is correlated with a lack of control over eating (r = 0.657) and excessive physical exercise to compensate for eating (r = 0.604). These values suggest a clear influence of stress on eating disorders during exam periods, supporting the hypothesis of a direct relationship between the two factors (see Table 7).

**Figure 3:**
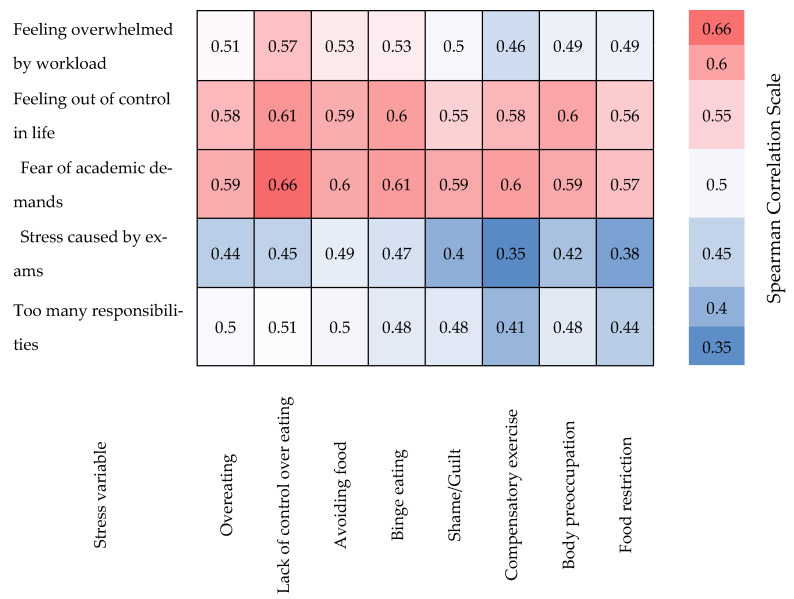
Heatmap of Spearman correlations between perceived academic stress and disordered eating behaviors. Note: Stronger correlations are represented by darker shades of red. All values are significant at *p* < 0.001. Correlation coefficients range from 0.35 (dark blue) to 0.66 (dark red).

To further enhance the interpretation of the results, we generated a heatmap to visually represent the Spearman correlations between perceived academic stress variables and disordered eating behaviors. This graphical representation allows a quick identification of the most intense associations and facilitates a clearer understanding of the data structure (see Figure 3).

To explore the relationship between perceived stress and disordered eating among students, we also applied linear regression to analyze the influence of stress on eating behaviors.

1. The regression between stress, the feeling of overwhelm due to workload (I_5), and eating excessively compared to the initial plan (I_15).

As shown in Table 8, the linear regression analysis revealed a significant relationship between feeling overwhelmed and excessive eating, with a B coefficient of 0.581 and a high statistical significance (*p* < 0.001). Approximately 24.6% of the variability in eating behavior can be explained by this type of stress (R^2^ = 0.246), and the ANOVA test (F = 102.613; *p* < 0.001) confirms the validity of the model. These results support the hypothesis that stress related to workload negatively impacts students’ eating habits.

2. The regression between difficulty in controlling important aspects of life (I_6) and lack of control over eating (I_16).

The linear regression results indicate a significant relationship between the difficulty in controlling important aspects of life (I_6) and the lack of control over eating (I_16), with a B coefficient of 0.680 and high statistical significance (*p* = 0.0001). The model explains 32.6% of the variability (R^2^ = 0.326), and the ANOVA test (F = 152.301) confirms the validity of the analysis (see Table 9). These results support the hypothesis that higher stress levels contribute to the development of unbalanced eating behaviors.

3. The regression between stress caused by exams (I_8) and episodes of binge eating (I_18).

The linear regression results support Hypothesis 2, that higher levels of stress are associated with an increased frequency of compulsive eating episodes. The coefficient B = 0.573 (*p* < 0.001) indicates a significant positive relationship between exam-related stress (I_8) and compulsive eating behavior (I_18) (see Table 10). The model accounts for 20.8% of the variance (R^2^ = 0.208), and the ANOVA test is significant (F = 82.939, *p* = 0.0001), confirming that stress is a relevant predictor of this eating pattern.

**Hypothesis** **3:** *Insomnia mediates the relationship between stress and disordered eating.*

We conducted the analysis in three main steps. First, we explored the relationship between stress and insomnia. Next, we looked at the direct effect of stress on disordered eating behaviors. In the final step, we introduced insomnia as a mediator to determine whether the effect of stress on eating patterns would decrease. This approach allowed us to better understand the role of insomnia in linking stress to changes in eating behavior.

1. Examining the relationship between perceived stress and insomnia.

The results reveal a significant relationship between the feeling of being overwhelmed and difficulty falling asleep (B = 0.701; R^2^ = 0.515; F = 334.018; *p* < 0.001), indicating that over half of the variance in insomnia can be explained by stress related to workload (see Table 11). This finding confirms that increased stress negatively affects students’ sleep quality.

2. Testing the direct relationship between stress and eating disorders.

The linear regression results reveal a significant positive relationship between the feeling of being overwhelmed and excessive eating (B = 0.581; R^2^ = 0.246; F = 102.613; *p* < 0.001, see Table 12). This suggests that nearly a quarter of the variance in unhealthy eating behavior can be attributed to stress related to workload, highlighting the clear impact of academic stress on students’ eating habits.

3. Examining the relationship between stress and eating disorders with insomnia as a mediator.

The analysis indicates that insomnia is a significant mediator in the relationship between academic stress and excessive eating. When insomnia is considered, stress no longer has a significant direct effect on eating behavior (B = 0.050, *p* = 0.483), while insomnia remains a strong predictor (B = 0.758, *p* < 0.001, see Table 13). Approximately 44% of the variance in excessive eating (R^2^ = 0.439) can be explained by the combination of stress and sleep issues. These results confirm Hypothesis 3, that insomnia mediates the relationship between stress and eating behavior.

**Hypothesis** **4:** *It is assumed that students experiencing higher levels of stress are more likely to report a combination of insomnia and eating disorders, in comparison to those with lower stress levels.*

To test Hypothesis 4, we applied the independent *t*-test, comparing students with high stress levels (above the mean score of 3.93 on the “feeling overwhelmed” variable) to those with low stress levels. The results indicate that students with high stress reported significantly higher scores for both insomnia and disordered eating behaviors, confirming the hypothesis that higher stress is associated with greater sleep difficulties and unbalanced eating behaviors.

**Figure 4:**
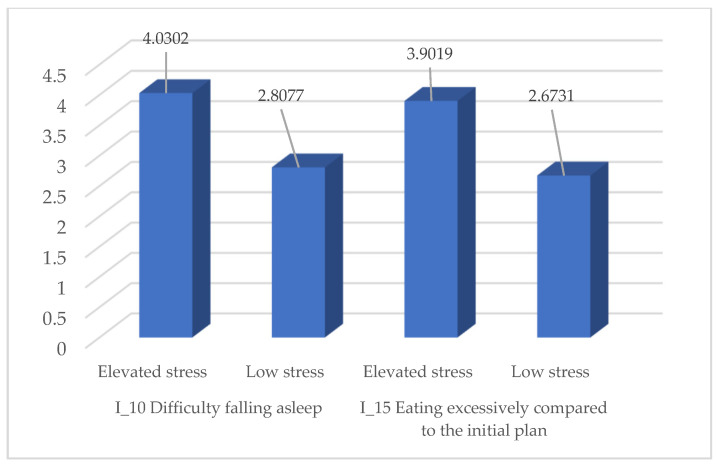
Mean scores of sleep difficulty and excessive eating by perceived stress level (elevated vs. low).

As shown in Table 14, students with elevated stress levels reported substantially higher mean scores on both difficulty falling asleep and excessive eating behaviors. These differences are visually summarized in Figure 4 below.

The results of the independent group samples *t*-test strongly support the hypothesis that students experiencing high levels of stress during exam periods report significantly higher scores in both insomnia and eating disorders compared to those with lower stress levels. Regarding insomnia, the difference between the two groups was highly significant (*t* = 11.516, *p* < 0.001), with a mean difference of 1.2225, indicating that students with high stress face greater difficulties in sleep initiation, maintenance, and achieving restful sleep. Also, for eating behavior, the differences between the two groups were significant (*t* = 10.931, *p* < 0.001), with a mean difference of 1.22881, indicating a higher prevalence of dysfunctional eating behaviors, such as compulsive eating or lack of control over eating, among students with high stress levels. Levene’s tests indicated unequal variances, and the analysis was adjusted accordingly (“equal variances not assumed”) These statistical results are summarized in Table 15.

These findings strengthen the conclusion that the level of perceived stress significantly affects students’ psychological and emotional well-being, influencing both sleep quality and eating habits. This highlights the importance of implementing intervention and support strategies for students during periods of high academic pressure.

## 5. Practical Implications and Recommendations

The findings of this study have important practical implications for improving student mental health support. The results show that academic stress, particularly during exam periods, can negatively affect both sleep and eating habits. As a result, universities should consider implementing preventive programs and counseling services aimed at helping students manage stress, recognize early signs of imbalance, and establish healthier daily routines. The recommendations primarily focus on promoting healthy practices, such as organizing workshops on sleep hygiene, balanced nutrition, and anxiety management techniques, ensuring easy access to psychological counseling services, and creating spaces dedicated to relaxation and stress relief, such as quiet rooms or recreational activities. Additionally, university professors could benefit from training aimed at recognizing signs of academic overload and fostering open communication with students. It is essential for educational institutions to prioritize mental health as a strategic concern, and the study’s findings can serve as a foundation for developing more inclusive educational policies focused on providing meaningful support to students during demanding academic periods.

## 6. Discussion

The results of this study confirm the strong associations between academic stress, sleep disturbances, and disordered eating behaviors among university students, especially during exam periods [29,30,31]. These findings are in line with previous research emphasizing the vulnerability of students under academic pressure and the multifactorial impact of stress on psychological well-being [32,33,34].

Perceived stress, particularly related to workload, deadlines, and perceived lack of control, was found to significantly correlate with symptoms of insomnia, such as difficulty falling asleep, unrefreshing sleep, and nighttime awakenings [35,36,37]. This aligns with studies highlighting the physiological mechanisms involved in stress-induced insomnia, including hyperactivation of the HPA axis and dysregulation of circadian rhythms [38,39,40]. In parallel, our findings confirm a robust association between academic stress and maladaptive eating behaviors, including compulsive eating, restrictive intake, and guilt after eating [41,42,43]. These behaviors are recognized as emotional coping mechanisms in response to chronic stress [44,45,46], as well as recent concerns regarding the influence of digital habits on student well-being [47,48,49,50,51,52,53,54,55,56,57,58,59,60,61]. The highest predictive value was identified for the relationship between the feeling of being overwhelmed and excessive food consumption beyond initial intentions, supporting the hypothesis that stress alters appetite regulation [62,63]. Importantly, insomnia was identified as a mediator in the relationship between perceived stress and disordered eating, suggesting that sleep disturbances may increase the risk of developing unhealthy eating patterns under stress [64,65,66].

This mediation effect has been previously discussed in studies exploring the bidirectional associations between insomnia and emotional dysregulation related to food [67,68,69]. Although the use of pharmacological solutions was limited, the elevated levels of psychological discomfort highlight the need for preventive strategies. Universities should implement interventions focused on stress management, sleep hygiene, and healthy eating education, along with improving access to psychological services [70,71,72].

Overall, the findings emphasize the necessity of institutional support systems to reduce the long-term impact of academic stress on students’ mental and physical health [73,74,75,76,77].

## 7. Limitations and Future Directions

Although this study provides meaningful insights into the relationship between academic stress, insomnia, and disordered eating behaviors among university students, certain limitations should be acknowledged. The cross-sectional design limits the ability to draw conclusions about causality, as all variables were assessed at a single moment in time. It remains possible that other unmeasured psychological or contextual factors may have contributed to the associations identified. Additionally, the reliance on self-reported questionnaires may introduce subjectivity, with participants potentially underestimating or overestimating their experiences of stress, sleep disturbance, or eating behaviors depending on their emotional state or interpretation of the items.

Despite these limitations, the study has several notable strengths. It included a relatively large sample (N = 317), which improves the generalizability of findings within the Romanian university student population. The use of validated psychometric tools, namely the Perceived Stress Scale (PSS), Insomnia Severity Index (ISI), and the Eating Disorder Examination Questionnaire (EDE-Q), strengthens the methodological rigor and ensures reliable measurement of key variables. Furthermore, data collection was conducted during an actual exam period, providing a realistic snapshot of students’ psychological functioning under academic pressure. The use of a non-probability convenience sampling method represents a key limitation, as it may introduce sampling bias and affect the generalizability of the findings. This potential bias should be acknowledged when interpreting the results. Moreover, the absence of objective assessment tools, such as clinical interviews, actigraphy, or food intake logs, reduces the depth of behavioral evaluation.

Another methodological limitation concerns the lack of control for sociodemographic variables. Although factors such as age, gender, and year of study were recorded and described, they were not included in the statistical analyses as potential covariates. Given the documented influence of these variables on stress, sleep, and eating behaviors, their exclusion may have affected the accuracy of the results. Future studies should consider incorporating multivariate models that adjust for sociodemographic differences to provide a more comprehensive and nuanced understanding of the observed associations.

The study’s focus on Romanian academic institutions may also restrict the applicability of the results to students from other cultural or educational contexts. Future research should build on these findings by employing longitudinal designs that allow for the exploration of causal pathways and the temporal evolution of stress, sleep disturbances, and disordered eating behaviors. It would be beneficial to implement and evaluate stress reduction interventions specifically targeting university students during exam sessions, with a focus on their effectiveness in preventing insomnia and maladaptive eating patterns. Further investigations might also examine the mediating role of sleep disturbances in the link between academic stress and eating dysfunctions to develop more targeted psychological interventions.

In addition to emphasizing the importance of stress reduction interventions, future programs should be grounded in evidence-based approaches. Specific interventions such as sleep hygiene education, cognitive and behavioral strategies for managing anxiety, or structured emotional regulation programs could be implemented at the university level. These targeted measures may help students build resilience and reduce the risk of stress-related sleep and eating disorders.

Moreover, previous research has highlighted how factors such as stress mindset, smartphone dependence, bedtime procrastination, and impulsive-compulsive traits can influence students’ vulnerability to stress-related disturbances in sleep and eating behaviors [78,79,80,81,82].

By identifying insomnia as a key mechanism through which stress influences eating behavior, this study contributes to a more nuanced understanding of students’ mental health risks during high-pressure academic periods. The results underscore the need for universities to prioritize accessible psychological support services and to promote proactive strategies that foster resilience and emotional well-being in student populations [83,84,85,86,87].

## 8. Conclusions

The conclusions of this study emphasize a significant relationship between perceived academic stress during exam periods, insomnia, and disordered eating behaviors among students. The results support the initial hypotheses, demonstrating that elevated stress levels are linked to a higher frequency of insomnia symptoms and maladaptive eating behaviors, including compulsive eating, restrictive intake, and a preference for unhealthy foods. Statistical analyses revealed significant positive correlations among these variables and identified insomnia as a mediating factor in the relationship between academic stress and eating behavior.

Perceived stress not only impairs sleep quality but also contributes to disrupted eating patterns, illustrating a vicious cycle involving psychological distress, sleep disturbances, and poor dietary habits. Through regression analysis and *t*-tests, the study identified clear differences between students with high and low stress levels, both in terms of sleep disruption and disordered eating.

Overall, the findings suggest that academic stress during exam periods has a substantial and multifaceted impact on student well-being. These outcomes underscore the need for continuous intervention from educational institutions, particularly through psycho-educational support, to prevent the escalation of such issues. Promoting a sustainable balance between academic demands and mental health should be regarded as a strategic priority in all educational settings.

## Figures and Tables

**Table 1:** Perceived level of academic stress during the exam sessions.

Descriptive Statistics	*n*	Min	Max	Mean	Std. Deviation
5. The feeling of overwhelm due to workload	317	1	5	3.9338	0.68346
6. The difficulty in controlling important aspects of life	317	1	5	3.9022	0.67027
7. The fear of not keeping up with academic demands	317	1	5	3.8801	0.69228
8. The stress caused by the exams	317	1	5	**3.9968**	0.63395
9. The feeling of being overwhelmed by too many responsibilities	317	1	5	3.9842	0.65825
Valid N (listwise)	317				

Note: Highest perceived stress value is highlighted in bold.

**Table 2:** Assessment of insomnia during the exam session.

Descriptive Statistics	*n*	Min	Max	Mean	Std. Deviation
10. Difficulty falling asleep	317	1	5	3.8297	0.66751
11. Difficulty staying asleep (waking up frequently)	317	1	5	3.7760	0.714
12. Perceiving your sleep as unrefreshing or insufficient	317	1	5	3.8612	0.65577
13. The impact of exam anxiety on sleep	317	1	5	3.8517	0.67042
14. Fatigue and exhaustion caused by lack of sleep	317	1	5	**3.8801**	0.63006
Valid N (listwise)	317				

Note: Highest insomnia-related score is highlighted in bold.

**Table 3:** Assessment of eating disorders during the exam session.

Descriptive Statistics	*n*	Min	Max	Mean	Std. Deviation
15. Eating excessively compared to the initial plan	317	1	5	3.7003	0.80057
16. Lack of control over eating	317	1	5	3.694	0.79818
17. Avoiding food in order to manage or reduce body weight	317	1	5	3.6877	0.83833
18. Episodes of binge eating	317	1	5	**3.7066**	0.79499
19. The feeling of shame or guilt after eating	317	1	5	3.653	0.89977
20. Excessive exercise to compensate for overeating	317	1	5	3.6183	0.88735
21. The concern about body weight and shape	317	1	5	3.6909	0.8262
22. Drastic food restriction to control weight	317	1	5	3.6404	0.88769
Valid N (listwise)	317				

Note: Highest disordered eating score is highlighted in bold.

**Table 4:** Relationship between stress, insomnia, and eating disorders during the exam session.

Descriptive Statistics	*n*	Min	Max	Mean	Std. Deviation
23. The contribution of exam session stress to insomnia	317	1	5	3.8486	0.69065
24. The impact of stress on eating behaviors	317	1	5	3.7729	0.71078
25. The connection between insomnia and binge eating	317	1	5	3.6625	0.86946
26. Reducing food intake or skipping meals due to stress	317	1	5	3.7603	0.79909
27. The effect of insomnia on the appetite and eating habits	317	1	5	3.7445	0.77194
28. The preference for unhealthy foods during stressful periods	317	1	5	**3.9306**	0.75152
29. Eating as a coping mechanism for stress	317	1	5	3.7224	0.80656
30. The frequency of binge eating episodes during exam sessions	317	1	5	3.7256	0.81738
31. The stress related to body image during exam sessions	317	1	5	3.7098	0.85184
32. Changes in body weight because of stress or insomnia	317	1	5	3.7476	0.76268
33. The frequency with which lack of sleep affects the ability to organize daily meals	317	1	5	3.8139	0.72918
34. Using food as a mechanism to calm the anxiety related to exams	317	1	5	3.7319	0.81948

Note: Highest combined factor score (stress, insomnia, and eating) is highlighted in bold.

**Table 5:** Use of sleep and stress medication during exams.

Descriptive Statistics	*n*	Min	Max	Mean	Std. Deviation
35. Use of sleep medications during exam sessions	317	1	5	1.8454	1.04249
36. Use of medication to reduce stress or anxiety	317	1	5	1.8454	0.99592
37. Using natural supplements to help with sleep	317	1	5	**1.8927**	1.06788
38. Taking prescribed medication to cope with stress and anxiety	317	1	5	1.3533	0.91822
39. Using sleep medication more frequently during exam time	317	1	5	1.8864	1.06425
Valid N (listwise)	317				

Note: Highest perceived usage is highlighted in bold.

**Table 6:** Correlations between stress level and insomnia (Spearman correlation).

Correlation Coefficient			
Stress Variable	Difficulty Falling Asleep (I_10)	Frequent Awakenings (I_11)	Unrefreshing Sleep (I_12)
I_5 Feeling overwhelmed by workload	r = **0.684** ** *p* < **0.001** N = 317	r = **0.642** ** *p* < **0.001** N = 317	r = **0.721** ** *p* < **0.001** N = 317
I_6 Feeling out of control in life	r = **0.703** ** *p* < **0.001** N = 317	r = **0.728** ** *p* < **0.001** N = 317	r = **0.716** ** *p* < **0.001** N = 317
I_8 Stress caused by exams	r = **0.682** ** *p* < **0.001** N = 317	r = **0.558** ** *p* < **0.001** N = 317	r = **0.689** ** *p* < **0.001** N = 317
I_9 Too many responsibilities	r = **0.678** ** *p* < **0.001** N = 317	r = **0.594** ** *p* < **0.001** N = 317	r = **0.690** ** *p* < **0.001** N = 317

** Correlation is significant at the 0.01 level (2-tailed). Note: Bolded values are statistically significant at the 0.01 level, using two-tailed Spearman correlation.

**Table 7:** Spearman correlations between perceived stress and eating disorders among students.

Stress Variable	I_15 Overeating	I_16 Lack of Control over Eating	I_17 Avoiding Food	I_18 Binge Eating	I_19 Shame/Guilt	I_20 Compensatory Exercise	I_21 Body Preoccupation	I_22 Food Restriction
I_5 Feeling overwhelmed by workload	r = 0.513 **	r = 0.568 **	r = 0.528 **	r = 0.525 **	r = 0.502 **	r = 0.463 **	r = 0.491 **	r = 0.492 **
I_6 Feeling out of control in life	r = 0.576 **	r = 0.613 **	r = 0.589 **	r = 0.604 **	r = 0.552 **	r = 0.579 **	r = 0.568 **	r = 0.558 **
I_7 Fear of academic demands	r = 0.590 **	r = 0.657 **	r = 0.595 **	r = 0.606 **	r = 0.593 **	r = 0.604 **	r = 0.593 **	r = 0.570 **
I_8 Stress caused by exams	r = 0.443 **	r = 0.448 **	r = 0.487 **	r = 0.470 **	r = 0.399 **	r = 0.345 **	r = 0.424 **	r = 0.379 **
I_9 Too many responsibilities	r = 0.504 **	r = 0.508 **	r = 0.499 **	r = 0.483 **	r = 0.480 **	r = 0.408 **	r = 0.481 **	r = 0.441 **

** Note: All correlations are significant at the 0.01 level (2-tailed, *p* < 0.001). Statistically significant values are highlighted in bold.

**Table 8:** The linear regression analysis regarding the influence of feeling overwhelmed by workload on excessive eating compared to initial intentions.

ANOVA ^a^								
Model		Sum of Squares	df	Mean Square		F	Sig.	
1	Regression	49.764	1	49.764		**102.613**	**0.000 ^b^**	
	Residual	152.766	315	0.485				
	Total	202.530	316					
Coefficients ^a^								
Model				Unstandardized Coefficients		Standardized Coefficients	*t*	Sig.
				B	Std. Error	Beta		
1	(Constant)			**1.416**	**0.229**		**6.189**	**0.000**
	I_5 The feeling of overwhelm due to workload			**0.581**	**0.057**	**0.496**	**10.130**	**0.000**

^a^ Dependent Variable: I_15 Eating excessively compared to the initial plan. ^b^ Predictors: (Constant) I_5 The feeling of overwhelm due to workload. Note: Statistically significant results are marked in bold.

**Table 9:** The linear regression analysis regarding the influence of the perception of difficulty in controlling important aspects of life on the lack of control over eating.

ANOVA ^a^							
Model		Sum of Squares	df	Mean Square	F	Sig.	
1	Regression	65.613	1	65.613	**152.301**	**0.000 ^b^**	
	Residual	135.706	315	0.431			
	Total	201.319	316				
Coefficients ^a^							
Model			Unstandardized Coefficients		Standardized Coefficients	*t*	Sig.
			B	Std. Error	Beta		
1	(Constant)		**1.041**	**0.218**		**4.774**	**0.000**
	I_6. The difficulty in controlling important aspects of life		**0.680**	**0.055**	**0.571**	**12.34**	**0.000**

^a^ Dependent Variable: I_16. Lack of control over eating. ^b^ Predictors: (Constant) I_6. The difficulty in controlling important aspects of life. Note: All bolded values are statistically significant (*p* < 0.001, two-tailed).

**Table 10:** Linear regression analysis on the impact of exam-related stress on episodes of compulsive eating.

ANOVA ^a^							
Model		Sum of Squares	df	Mean Square	F	Sig.	
1	Regression	41.625	1	41.625	**82.939**	**0.000 ^b^**	
	Residual	158.091	315	0.502			
	Total	199.716	316				
Coefficients ^a^							
Model			Unstandardized Coefficients		Standardized Coefficients	*t*	Sig.
			B	Std. Error	Beta		
1	(Constant)		**1.418**	**0.254**		**5.576**	**0.000**
	I_8 The stress caused by the exams		**0.573**	**0.063**	**0.457**	**9.107**	**0.000**

^a^ Dependent Variable: I_18 Episodes of binge eating. ^b^ Predictors: (Constant) I_8 The stress caused by the exams. Note: Bold values indicate statistically significant results (*p* < 0.001, two-tailed).

**Table 11:** Linear regression analysis of the influence of overwhelm due to workload on difficulty falling asleep.

ANOVA ^a^							
Model		Sum of Squares	df	Mean Square	F	Sig.	
1	Regression	72.464	1	72.464	**334.018**	**0.000 ^b^**	
	Residual	68.338	315	0.217			
	Total	140.801	316				
Coefficients ^a^							
Model			Unstandardized Coefficients		Standardized Coefficients	*t*	Sig.
			B	Std. Error	Beta		
1	(Constant)		**1.073**	**0.153**		**7.013**	**0.000**
	I_5 The feeling of overwhelm due to workload		**0.701**	**0.038**	**0.717**	**18.276**	**0.000**

^a^ Dependent Variable: I_10. Difficulty falling asleep. ^b^ Predictors: (Constant) I_5 The feeling of overwhelm due to workload. Note: Bolded values indicate statistically significant results (*p* < 0.001, two-tailed).

**Table 12:** Linear regression analysis regarding the influence of the feeling of being overwhelmed by workload on excessive eating behavior compared to initial intentions.

ANOVA ^a^							
Model		Sum of Squares	df	Mean Square	F	Sig.	
1	Regression	49.764	1	49.764	**102.613**	**0.000 ^b^**	
	Residual	152.766	315	0.485			
	Total	202.530	316				
Coefficients ^a^							
Model			Unstandardized Coefficients		Standardized Coefficients	*t*	Sig.
			B	Std. Error	Beta		
1	(Constant)		**1.416**	**0.229**		**6.189**	**0.000**
	I_5 The feeling of overwhelm due to workload		**0.581**	**0.057**	**0.496**	**10.13**	**0.000**

^a^ Dependent Variable: I_15 Eating excessively compared to the initial plan. ^b^ Predictors: (Constant) I_5 The feeling of overwhelm due to workload. Note: Bold values indicate statistically significant results (*p* < 0.001, two-tailed).

**Table 13:** Multiple linear regression analysis regarding the simultaneous influence of difficulty falling asleep and the feeling of being overwhelmed on excessive eating behavior relative to initial intentions.

ANOVA ^a^								
Model		Sum of Squares	df	Mean Square		F	Sig.	
1	Regression	88.978	2	44.489		**123.022**	**0.000 ^b^**	
	Residual	113.552	314	0.362				
	Total	202.530	316					
Coefficients ^a^								
Model				Unstandardized Coefficients		Standardized Coefficients	*t*	Sig.
				B	Std. Error	Beta		
1	(Constant)			**0.603**	**0.212**		**2.838**	**0.005**
	I_5 The feeling of overwhelm due to workload			0.050	0.071	0.043	0.702	0.483
	I_10 Difficulty falling asleep			**0.758**	**0.073**	**0.632**	**10.413**	**0.000**

^a^ Dependent Variable: I_15 Eating excessively compared to the initial plan. ^b^ Predictors: (Constant) I_10 Difficulty falling asleep, I_5 The feeling of overwhelm due to workload. Note: Bolded values indicate statistically significant results.

**Table 14:** Descriptive statistics regarding the difficulty of falling asleep and excessive eating behavior based on perceived stress levels.

Group Statistics	Group_Stress	*n*	Mean	Std. Deviation	Std. Error Mean
I_10 Difficulty falling asleep	Elevated stress	265	**4.0302**	0.42533	0.02613
	Low stress	52	**2.8077**	0.74198	0.10289
I_15 Eating excessively compared to the initial plan	Elevated stress	265	**3.9019**	0.63797	0.03919
	Low stress	52	**2.6731**	0.75980	0.10537

Note: Bold values indicate statistically significant differences between high-stress and low-stress groups.

**Table 15:** Results of the independent samples *t*-test regarding the differences in difficulty falling asleep and excessive eating between high and low stress level groups.

Independent Samples Test		Levene’s Test for Equality of Variances		*t*-Test for Equality of Means						
		F	Sig.	*t*	df	Sig. (2-Tailed)	Mean Difference	Std. Error Difference	95% Confidence Interval of the Difference	
									Lower	Upper
I_10 Difficulty falling asleep	Equal variances assumed	**32.545**	**0.000**	**16.427**	**315**	**0.000**	**1.22250**	**0.07442**	**1.07607**	**1.36892**
	Equal variances not assumed			11.516	57.743	0.000	1.22250	0.10616	1.00998	1.43502
I_15 Eating excessively compared to the initial plan	Equal variances assumed	**11.690**	**0.001**	**12.290**	**315**	**0.000**	**1.22881**	**0.09999**	**1.03209**	**1.42553**
	Equal variances not assumed			10.931	65.844	0.000	1.22881	0.11242	1.00435	1.45327

Note: Bolded values represent statistically significant results (*p* < 0.001, two-tailed).

## Data Availability

The data used and/or analyzed during the current study are available from the corresponding author upon reasonable request. Unfortunately, these data are not publicly available because of privacy or ethical restrictions.

## Abbreviations

The following abbreviations are used in this manuscript:

SPSS	Statistical Package for the Social Sciences
ISI	Insomnia Severity Index
PPS	Perceived Stress Scale
EDE-Q	Eating Disorder Examination Questionnaire
SD	standard deviation
